# A familial congenital heart disease with a possible multigenic origin involving a mutation in *BMPR1A*

**DOI:** 10.1038/s41598-019-39648-7

**Published:** 2019-02-27

**Authors:** Till Joscha Demal, Melina Heise, Benedikt Reiz, Deepika Dogra, Ingrid Brænne, Hermann Reichenspurner, Jörg Männer, Zouhair Aherrahrou, Heribert Schunkert, Jeanette Erdmann, Salim Abdelilah-Seyfried

**Affiliations:** 1 Institute for Cardiogenetics, University Heart Centre Lübeck, University of Lübeck, DZHK (German Research Centre for Cardiovascular Research) partner site Hamburg/Lübeck/Kiel, D-23562 Lübeck, Germany; 2 Department of Cardiovascular Surgery, University Heart Centre Hamburg, D-20246 Hamburg, Germany; 3 Institute of Molecular Biology, Hannover Medical School, D-30625 Hannover, Germany; 4 Institute of Biochemistry and Biology, Potsdam University, D-14476 Potsdam, Germany; 5 Center for Public Health Genomics, University of Virginia, Charlottesville, VA 22903 USA; 6 Institute of Anatomy and Embryology, UMG, Göttingen University, D−37075 Göttingen, Germany; 7 Department of Cardiovascular Diseases, German Heart Centre Munich, Technical University of Munich (TUM) and DZHK (German Research Centre for Cardiovascular Research) partner site, D-80636 Munich, Germany

## Abstract

The genetics of many congenital heart diseases (CHDs) can only unsatisfactorily be explained by known chromosomal or Mendelian syndromes. Here, we present sequencing data of a family with a potentially multigenic origin of CHD. Twelve of nineteen family members carry a familial mutation [NM_004329.2:c.1328 G > A (p.R443H)] which encodes a predicted deleterious variant of BMPR1A. This mutation co-segregates with a linkage region on chromosome 1 that associates with the emergence of severe CHDs including Ebstein’s anomaly, atrioventricular septal defect, and others. We show that the continuous overexpression of the zebrafish homologous mutation *bmpr1aa*^*p*.*R438H*^ within endocardium causes a reduced AV valve area, a downregulation of Wnt/ß-catenin signalling at the AV canal, and growth of additional tissue mass in adult zebrafish hearts. This finding opens the possibility of testing genetic interactions between *BMPR1A* and other candidate genes within linkage region 1 which may provide a first step towards unravelling more complex genetic patterns in cardiovascular disease aetiology.

## Introduction

Congenital heart diseases (CHDs) are the most common organ malformations and affect 1% of newborns^1,2^. Due to recent improvements in the treatment of CHDs, increasing numbers of patients reach a reproductive age. This has raised renewed interest in understanding the molecular causes of CHDs with the aim of improving diagnostic or therapeutic tools. Although a variety of genes has been implicated in the development of CHDs, only a minority of these diseases is caused by monogenic mutations^3^. Hence, one of the most urgent challenges in cardiovascular disease aetiology is a better understanding of more complex genetic traits leading to CHDs.

A large proportion of all CHDs affect the formation of atrioventricular (AV) valves. In higher vertebrates, the endocardial cushions are precursors of AV valves, cardiac septa, and parts of the cardiac outflow tract. The atrioventricular endocardial cushions are formed by endocardial cells of the atrioventricular canal (AVC) that hypertrophy and migrate into the extracellular matrix in between the inner endocardial and the outer myocardial layer of the heart tube^4^. This process is known as endothelial-mesenchymal transition (endoMT). Afterwards the endocardial cushions located in the AVC form the atrioventricular valves^5^. Defective development of the endocardial cushions can lead to CHDs including atrial, ventricular, and atrioventricular septal defects in mice^6,7^.

The zebrafish is an excellent vertebrate model for functional studies of valve leaflet morphogenesis^8^. The zebrafish and human genome share a high degree of similarity with 69% of protein-coding zebrafish genes being related to genes found in humans^9^. Hence, the analysis of human congenital defects is feasible in this animal model. In contrast to human anatomy, the zebrafish heart consists of only one atrium and ventricle. These two cardiac chambers are separated by an AV valve. During zebrafish cardiac valve development, cardiac cushions elongate and form paired primitive bicuspid valve leaflets, which protrude from either side of the AVC into the lumen^10,11^. Within three months, the initially bicuspid valves transform into quadricuspid structures^12^.

The bone morphogenetic protein (BMP) pathway plays an important role in the development of embryonic heart valves^7,13,14^. BMPs are involved in the development of endocardial cushions via endoMT, the maturation of the tissue surrounding the AV valves, and the septation of heart cavities^14^. In mice, expression of *BMP-Receptor 1A* (*BMPR1A*, *also known as ALK3*) is required in both endocardium and myocardium to ensure the correct development of endocardial cushions^15,16^.

There are numerous case reports about patients with *BMPR1A* mutations and cardiac septal defects. These defects often occur in the context of deletion syndromes and are in combination with mental retardation, facial dysmorphism, or juvenile polyposis syndrome (JPS)^17–20^. In addition, isolated *BMPR1A* mutations have been reported to associate with cardiac malformations and occurrence of JPS. Several missense mutations of *BMPR1A* are associated with the emergence of ventricular septal defects and Ebstein’s anomaly^21^. Mutations in the BMP pathway have also been connected to non-syndromic CHDs. D’Allessandro *et al*. described three rare mutations of *BMPR1A* (p.R478H, p.D429V, and p.P481S) and the concomitant occurrence of atrioventricular septal defects^22^.

The involvement of *BMPR1A* in the development of Ebstein’s anomaly has also been shown in animal studies. Mice with a conditional knockout of *BMPR1A* in the AV canal displayed a malformation of the tricuspid valve and a disruption of the annulus fibrosus with a consecutive ventricular preexcitation, both which are characteristics of Ebstein’s anomaly^23^.

Although numerous reports of patients with *BMPR1A* mutations and associated CHDs exist, a clear causal connection has not yet been demonstrated in functional studies. Since chromosomal and Mendelian syndromes explain only 20% of the cases^24^, also more complex genetic processes may have an important influence on the development of CHD.

In 1997, we described a family with multiple cardiac cushion defects (e.g. Ebstein’s anomaly, atrioventricular septal defect, and aortic stenosis)^25^. Within four generations, at least 13 family members were affected. Here we present the results of next generation sequencing of this family. Using an adult zebrafish model, we provide a detailed functional analysis of a candidate mutation in *BMPR1A*. Our results indicate a more complex genetic trait involved in CHD.

## Results

### Phenotype and genotype data of a large pedigree with CHDs

**Figure 1:**
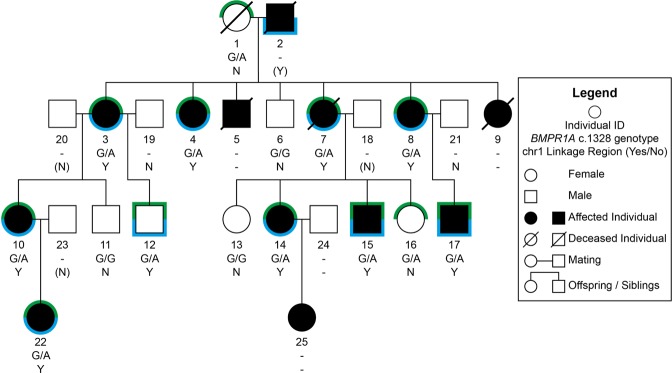
Pedigree of the affected family. Green marks indicate that the individual carries the BMPR1A mutation and blue marks represent the co-segregation of the chromosome 1 linkage region. For each individual, personal ID, *BMPR1A*^*c.1328*^ genotype, and co-segregation status of the chromosome 1 linkage region is listed. G/A: Heterozygote carrier of *BMPR1A*^*p.R443H*^. G/G: No mutation. ‘-’: No sequencing data available. N: No; Y: Yes. Values in brackets are based on the haplotype.

Since the first description by Schunkert *et al*. of a family as “a large pedigree with valvuloseptal defects^25^”, two more family members have been identified with 13 of 19 family members suffering from CHDs (Fig. 1). No extracardiac anomalies were reported, apart from a single patient that anamnestically suffered from “severe malformations” before he died during the first days after birth (no medical records available). The main phenotypes were characterized by atrioventricular septal defects (AVSD) (n = 3), an atrial septal defect (ASD) (n = 1), a ventricular septal defect (VSD) (n = 1), Ebstein’s anomalies (n = 4), Wolff-Parkinson-White (WPW) syndromes (n = 3), cleft mitral valves (n = 3), and right bundle branch blocks (RBBB) (n = 3) (see Supplementary Table S1 for a summary of all available clinical features and patient details).

The family was first characterized by short tandem repeat (STR)-based linkage analysis^26^. Although this led to the discovery of a linked region on chromosome 1, the signal did not yield genome-wide significance based on an established logarithm of the odds (LOD)-score threshold of 3. Given the large region and the limitations in sequencing technologies at that time, the analysis rested until, almost two decades later, we reanalysed the family by current state-of-the-art linkage analysis techniques combined with genome and exome sequencing.

**Figure 2:**
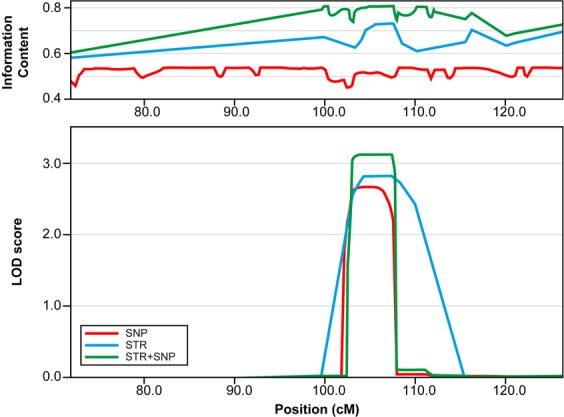
Chromosome 1 linkage region based on SNP and STR data. SNP data (red): information content ~0.5, peak LOD score 2.663 at position 104.823–105.289 cM (80675529–81258943 bp); STR data (blue): information content ~0.6–0.7, peak LOD score 2.817 at position 106.85–107.4 cM (81998598–82895464 bp). Combination of SNP and STR data (green): information content ~0.6–0.8, peak LOD score 3.118 at position 106.666–107.4 cM (81911788–82895464 bp).

We combined the STR data from candidate linkage regions with dense SNP markers analysis. STR data was available for individuals 1, 3, 4, 6–8, 10, 11, 13–17, 19, and 21. SNP data was available for the individuals 3, 4, 8, 10–13, 15, 17, and 22 (Fig. 1). In total, five distinct linkage signals on chromosomes 1, 2, 4, 10, and X were detected by a nonparametric linkage analysis (Supplementary Fig. S1). Based on the combined STR and SNP data, the only signal which exceeded a LOD score of 3 was on chromosome 1 (Fig. 2). The LOD score was above 3 at genomic position hg19 chr1: 77887835–83041391 bp and overlaps with the RefSeq genes *ADGRL2*, *ADGRL4*, *AK5*, *DNAJB4*, *FUBP1*, *GIPC2*, *IFI44*, *IFI44L*, *LINC01781*, *LOC101927412*, *LOC101927434*, *MGC27382*, *MIGA1*, *NEXN*, *PTGFR*, *USP33*, and *ZZZ3*.

All of the 10 CHD affected family members that were available for sequencing analysis carry that linkage region (Fig. 1). The odds ratio (OR) is significantly higher than 1 (p = 0.0001, Fisher’s exact test). One unaffected family member (Individual No. 12) also carries the linkage region, which suggests reduced penetrance, a multifactorial inheritance, and/or the contribution of environmental factors.

After the identification of potential disease-associated loci by linkage analysis, we screened the chromosome 1 region in more detail with the aim to identify candidates for disease-causing variants. However, a clear pathogenic variant was not identified within the coding regions of genes in the linkage region on chromosome 1. Due to limited DNA availability, not all family members were sequenced.

One variant that strongly co-segregated with linkage region 1 was within the *BMPR1A* locus on chromosome 10 (hg19 genomic position: chr10:g.88681438 G > A). This variant causes a predicted deleterious AA-change [NM_004329.2:c.1328 G > A(p.(R443H)] as indicated by multiple functional prediction tools including SIFT^27^, PolyPhen2^28^, MutationTaster2^29^, CADD^30^, and DANN^31^. So far, this variant has only been reported in the ClinVar registry describing an association to the Hereditary cancer-predisposing syndrome^32^. Subsequent validation of this variant in other family members revealed, that the co-segregation is not perfect: although all affected family members carried the variant, it was also present in three unaffected family members (Fig. 1; No. 1, 12, and 16). However, the variant occurs significantly more frequently in family members suffering from CHD when compared to their unaffected relatives (p = 0.044, Fisher’s exact test).

Because *BMPR1A*^*p*.*R443H*^ (chromosome 10) and the linkage region on chromosome 1 showed a strong co-segregation within the family, we next tested whether a chromosomal translocation was present. However, this possibility was excluded by Fluorescence *in situ* hybridization (FISH) analysis.

### The human *BMPR1A*^*p*.*R443H*^ mutation complements the loss of zebrafish Bmpr1aa/Bmpr1ab receptors and hence encodes a functional receptor variant

To test whether the human gene encoding BMPR1A^p.R443H^ is functional, we performed a complementation assay based on mRNA injections into zebrafish *bmpr1aa*/*ab* double morphants. Loss of the two Bmpr1a proteins in zebrafish causes severe dorsalization defects that can partially be rescued by the injection of human *BMPR1A*^*WT*^ mRNA^33^. In addition to using human *BMPR1A*^*WT*^ and *BMPR1A*^*p*.*R443H*^ mRNAs, we also injected *BMPR1A*^*p*.*L342R*^ (“Linkspoot”) mRNA that contains a missense mutation which produces a dominant-negative BMPR1A^34^. Finally, we also tested *BMPR1A*^*p*.*R443C*^ that harbours a mutation, which is associated with the occurrence of juvenile polyposis syndrome (JPS)^35^. As *BMPR1A*^*p*.*R443C*^ (referred to as JPS variant) affects the same residue as the mutation found in the reported family, we used this mutant variant to elucidate possible residue-specific effects on the BMPR1A mutant protein (see Supplementary Table S2 for a list of all *BMPR1A* variants used in this study; Supplementary Fig. S2 shows a sequence alignment of human BMPR1A with zebrafish Bmpr1aa).

**Figure 3:**
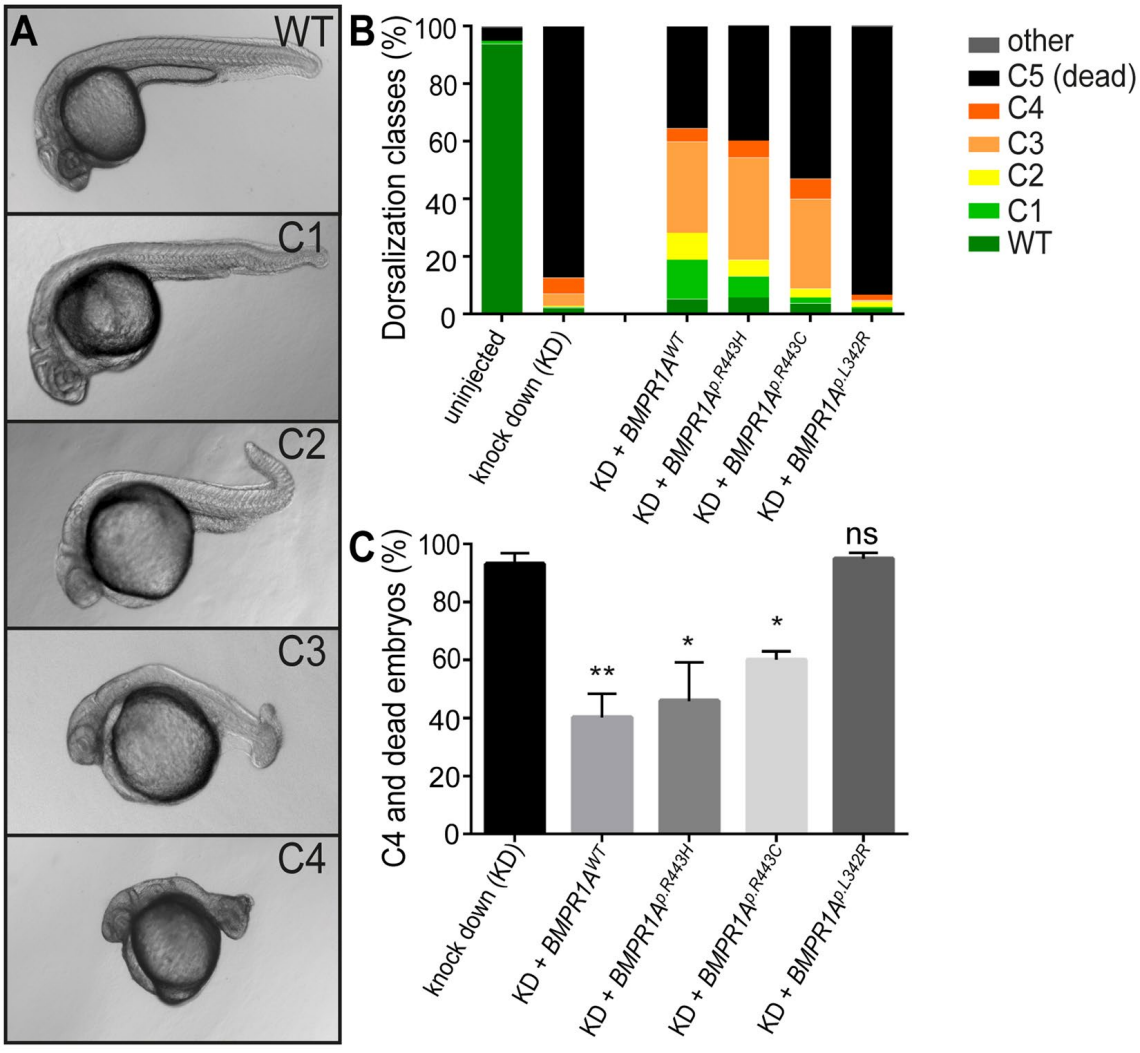
Rescue of bmpr1aa morphant dorsalization phenotypes in zebrafish by injection of mRNA encoding human BMPR1A. (**A**) Representative classes of dorsalization phenotypes in zebrafish embryos at 24hpf as previously described^36^. (**B**) Mean percentages of the five dorsalization classes among the different experimental groups [total number of embryos analysed: uninjected, n = 528; bmpr1aa MO knockdown (KD), n = 217; KD + *BMPR1A*^*WT*^ mRNA, n = 228; KD + *BMPR1A*^*p.R443H*^ mRNA, n = 255; KD + *BMPR1A*^*p.R443C*^ mRNA (JPS), n = 147; KD + *BMPR1A*^*p.L342R*^ mRNA (Linkspoot), n = 219]. (**C**) Share of embryos with C4 phenotype or lethality (mean ± SEM) after knockdown and human *BMPR1A* mRNA rescue injections by 24hpf [Total number of injected embryos: n = 1066 (Supplementary Tables S6, S7, S8, S9, S10). Number of experiments: KD, n = 5; KD + *BMPR1A*^*WT*^ mRNA, n = 6; KD + *BMPR1A*^*p.R443H*^ mRNA, n = 5; KD + *BMPR1A*^*p.R443C*^ mRNA (JPS), n = 4; KD + *BMPR1A*^*p.L342R*^ mRNA (Linkspoot), n = 4. Indicated is the statistical significance of the difference between the occurrence of severe dorzalisation defects in each condition compared with the pure knockdown (KD) (*p’ < 0.05, **p’ < 0.01, ns: not significant)].

For complementation assays, we co-injected *BMPR1A* mRNAs together with antisense oligonucleotide morpholinos (MO) against *bmpr1aa* and *bmpr1ab*. At 24hpf, zebrafish embryos were classified into one of four different dorsalization classes that were categorized from C1 to C4 with ascending severity as previously described^36^ (Fig. 3A).

As previously shown, co-injection of *BMPR1A*^*p*.*L342R*^ (“Linkspoot”) mRNA together with *bmpr1aa/ab* MOs did not rescue the morphant phenotype^34^. In comparison, all other mRNAs showed a statistically significant rescue (Fig. 3C). Hence, contrary to our expectations based on the functional prediction tools used in this study, human BMPR1A^p.R443H^ and BMPR1A^p.R443C^ (JPS variant) receptor variants are functional in zebrafish and functionally complement the Bmpr1aa/ab knockdown-associated dorsalization phenotypes in zebrafish embryos. This finding provided further evidence for a genetic pattern of inheritance associated with the CHDs that is more complex than initially assumed.

### The diameter of zebrafish embryonic AV valves is not affected by pan-endothelial/endocardial overexpression of a *bmpr1aa*^*p*.*R438H*^ mutant

Bmp signalling has an essential role during valve development in mice, where it controls endoMT^16^. In zebrafish, *bmp4* is highly expressed at the cardiac cushions and Bmp receptors including Bmpr1aa are expressed within the early endocardium^37^. To elucidate whether a zebrafish Bmpr1aa^p.R438H^ variant corresponding to the human BMPR1A^p.R443H^ variant exerts some dominant-negative or gain-of function activity which may not be detected during dorsoventral pattern formation of the zebrafish embryo, we next analysed its activity during zebrafish cardiac valve leaflet formation. To this end, we generated two stable transgenic lines of zebrafish for Gal4-dependent overexpression of *bmpr1aa*^*p*.*R438H*^ [*Tg(UAS:bmpr1aa*^*p*.*R438H*^*_IRES_EGFP)*] or *bmpr1aa*^*WT*^ variants [*Tg(UAS:bmpr1aa*^*WT*^*_IRES_EGFP)*]. We used these stable transgenic lines in combination with the pan-endothelial activator line *Tg(fli1a:GAL4FF)*^*ubs3* ^^38^ to drive expression specifically within endocardium/endothelium. We validated that expression from these transgenic overexpression lines was detectable within endothelium, by performing whole mount *in situ* hybridizations using an *EGFP* probe against the bicistronic *bmpr1aa*_IRES_*EGFP* mRNAs (Supplementary Fig. S3).

Functional studies of early cardiac development revealed that three independent stable transgenic lines carrying the mutation (allele numbers md60, md61, and md66) were phenotypically not distinguishable. Similarly, two stable transgenic lines with the WT version of the receptor gene (allele numbers md65 and md67) did not show any phenotypes.

**Figure 4:**
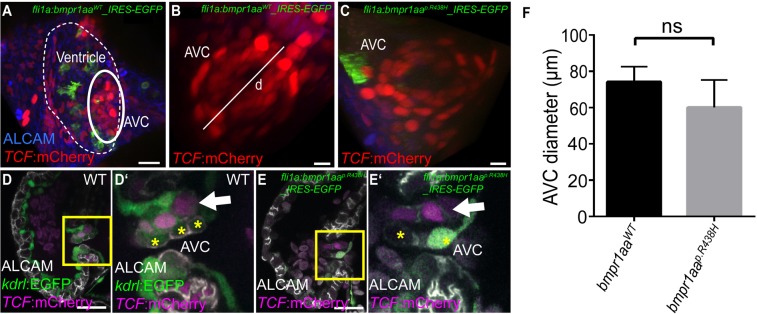
Pan-endothelial expression of a zebrafish Bmpr1aa^p.R438H^ mutant protein does not affect embryonic valvulogenesis. (**A**–**E**) Shown are reconstructions of confocal z-stack images of the zebrafish embryonic atrioventricular canal (AVC) region. (**A**) Embryonic cardiac ventricle in a *Tg(fli1a:Gal4FF)*^*ubs3*^; *Tg(UAS:bmpr1aa*^*WT*^*_IRES_EGFP)*^*md65*^; *Tg(7xTCF-Xia.Sia:NLS-mCherry)*^*ia5*^ zebrafish at 120hpf. The AVC region is outlined. Scale bar = 20 µm. (**B**) AVC region in a *Tg(fli1a:Gal4FF)*^*ubs3*^; *Tg(UAS:bmpr1aa*^*WT*^*_IRES_EGFP)*^*md65*^; *Tg(7xTCF-Xia.Sia:NLS-mCherry)*^*ia5*^ or (**C**) *Tg(fli1a:Gal4FF)*^*ubs3*^; *Tg(UAS:bmpr1aa*^*p.R438H*^*_IRES_EGFP)*^*md60*^; *Tg(7xTCF-Xia.Sia:NLS-mCherry)*^*ia5*^ zebrafish at 120hpf. Indicated is the diameter of the AVC (d). Scale bar = 10 µm. (**D**,**D′**) Embryonic valve formation in the WT zebrafish embryo at 72hpf. Box indicates the region shown in (**D′**). Valve leaflets in WT embryos are characterized by double-layering with an abluminal population of AVC cells that has active Wnt signalling marked by *Tg(7xTCF-Xia.Sia:NLS-mCherry)*^*ia5*^ (arrow). Cell membranes of luminal cells are marked by immuno-labelling against ALCAM (cells marked by asterisks). (**E**,**E′**) Similarly, valvulogenesis is not affected in *Tg(fli1a:Gal4FF)*^*ubs3*^; *Tg(UAS:bmpr1aa*^*p.R438H*^*_IRES_EGFP)*^*md60*^; *Tg(7xTCF-Xia.Sia:NLS-mCherry)*^*ia5*^ embryos that show a normal double-layered leaflet morphology with Wnt signalling in abluminal cells (arrow) and ALCAM-positive luminal cells (asterisks). Scale bars = 20 µm. (**F**) The diameter of the AVC in zebrafish embryos with pan-endothelial overexpression of *bmpr1aa*^*p.R438H*^ does not significantly differ from that upon *bmpr1aa*^*WT*^ overexpression [total number of embryos analysed: *bmpr1aa*^*WT*^, n = 6; *bmpr1aa*^*p.R438H*^, n = 8; two-sided student***’***s t-test, p = 0,066].

In functional tests, we compared valve size and morphology in *bmpr1aa*^*p*.*R438H*^ or *bmpr1aa*^*WT*^-overexpressing zebrafish embryos at 120hpf. At that stage, the AVC diameter was marked by the transgenic Wnt signalling reporter line *Tg(7xTCF-Xla*.*Sia:NLS-mCherry*)^*ia5*^ which strongly labels valve leaflets^39^ (Fig. 4A). The maximum AVC diameter was measured from edge to edge of the labelled valve leaflets (Fig. 4B). However, the AVC diameters of embryos expressing either of the two *bmpr1aa* variants did not significantly differ (*bmpr1aa*^*p*.*R438H*^: 60.2 ± 15.1 µm, n = 8; *bmpr1aa*^*WT*^: 74.1 ± 8.4 µm, n = 6; two-sided student’s t-test, p = 0.066, Fig. 4F). Obvious morphological changes due to the overexpression of *bmpr1aa*^*p*.*R438H*^ were not detectable (Fig. 4D,E). Hence, the endocardial/endothelial expression of *bmpr1aa*^*p*.*R438H*^ does not obviously interfere with growth or morphology of zebrafish AV valves during embryogenesis.

### Adult zebrafish expressing the *bmpr1aa*^*p*.*R438H*^ variant have smaller AV valves and reduced Wnt signalling within adult valve leaflets

**Figure 5:**
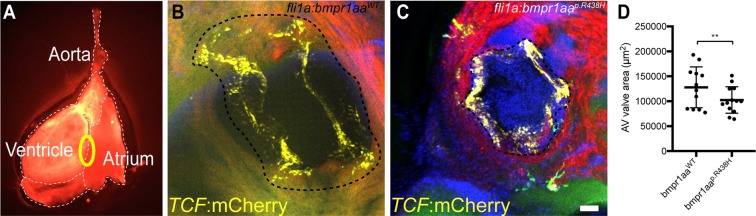
Adult zebrafish with pan-endothelial expression of Bmpr1aa^p.R438H^ have smaller AV valves. (**A)** Fluorescence microscopy image of an adult zebrafish heart (ventral view). Circle indicates the position of the AV valve. **(B**,**C)** Confocal z-stack maximum intensity projection of adult AV valves of zebrafish with (**B**) *Tg(fli1a:Gal4FF)*^*ubs3*^; *Tg(UAS:bmpr1aa*^*WT*^*_IRES_EGFP)*^*md65*^; *Tg(7xTCF-Xia*.*Sia:NLS-mCherry)*^*ia5*^ or (**C**) *Tg(fli1a:Gal4FF)*^*ubs3*^; *Tg(UAS:bmpr1aa*^*p*.*R438H*^*_IRES_EGFP)*^*md60*^; *Tg(7xTCF-Xia*.*Sia:NLS-mCherry)*^*ia5*^. The interrupted line indicates the AV valve annulus. This valve annulus surrounds the edge of the valve leaflets marked by TCF expression and occurs in the confocal images as a black ring. The area bounded by the AV valve annulus was measured to quantify the valve size. The AV valve measurement technique is further explained in Supplementary Fig. S4. Scale bar = 50 µm. **(D)** Scatter plot of the AV valve area measurements. Adult zebrafish overexpressing Bmpr1aa^p.R438H^ show a significant reduction in AV valve area [total number of embryos analysed: *bmpr1aa*^*WT*^, n = 12; *bmpr1aa*^*p*.*R438H*^, n = 13; ANCOVA, F(1, 22) = 10.73; **p = 0.003].

During growth of zebrafish heart, the initially bicuspid AV valves undergo some remodelling to form the mature quadricuspid valves^12,40^. However, the precise timing and mechanism of this remodelling process is still unknown. To analyse the potential impact of the mutant Bmpr1aa variant on this valve remodelling process, we raised zebrafish to adulthood that were continuously expressing either *bmpr1aa*^*p*.*R438H*^ or *bmpr1aa*^*WT*^ within endocardial/endothelial cells. We also employed the transgenic Wnt reporter line *Tg*(*7xTCF-Xla*.*Sia:NLS-mCherry*)^*ia5*^ which strongly labels valve leaflets to identify these structures within the adult heart^12,39^. After raising zebrafish for a period of 3–5 months under conditions of continuous endocardial/endothelial overexpression of *bmpr1aa*^*p*.*R438H*^ or *bmpr1aa*^*WT*^, the AV valvular morphology consisting of four valve leaflets was not affected. Hence, the basic transition from bicuspid to quadricuspid valves occurred normally. To characterize the AV valve morphology in more detail, we extracted transgenic zebrafish hearts and carefully severed the atrium from the ventricle which exposes the AV valve (Fig. 5A). Quantifications of 3D confocal z-stacks of adult zebrafish AV valves revealed, that the area bounded by the AV valve annulus (Fig. 5B,C) was significantly reduced in animals with a continuous endothelial-specific overexpression of the *bmpr1aa*^*p*.*R438H*^ mutant when compared to the control fish that were overexpressing *bmpr1aa*^*WT*^ (*bmpr1aa*^*p*.*R438H*^: 94,638.6 µm^2^, n = 13; *bmpr1aa*^*WT*^: 136,352.7 µm^2^, n = 12; ANCOVA; F(1, 22) = 10.73, p = 0.003; Fig. 5D; Supplementary Fig. S4). Since valve morphogenesis is affected by the size of the developing larvae^41^, an analysis of covariance (ANCOVA) was used, which statistically removes the effects of fish length on AV valve area using linear regression before performing a standard ANOVA. The AV valve measurement technique is further explained in Supplementary Fig. S4.

Canonical Wnt signalling acts as a mitogenic trigger of cushion mesenchyme proliferation following endoMT^42^ and the inhibition of Wnt/β-catenin signalling induces a lack of endocardial cushion tissue^43^. During zebrafish embryonic cardiac valve leaflet morphogenesis, the Wnt reporter is expressed on the abluminal side of the forming valve leaflet^10,11^. Strikingly, we found that adult fish overexpressing *bmpr1aa*^*p*.*R438H*^ within endocardium displayed a severe reduction of *Tg*(*7xTCF-Xla*.*Sia:NLS-mCherry*)^*ia5*^ reporter expression in comparison to fish overexpressing *bmpr1aa*^*WT*^ (Supplementary Fig. S4). This finding is indicative of a decreased Wnt signalling activity within adult AV valves upon *bmpr1aa*^*p*.*R438H*^ overexpression. Taken together, these findings demonstrate that the pan-endothelial overexpression of *bmpr1aa*^*p*.*R438H*^ causes a reduced AV valve size in adult zebrafish that may be triggered by a downregulation of Wnt/β-catenin signalling.

### Ectopic valvular tissue mass occurs in adult zebrafish with an endocardial overexpression of the *bmpr1aa*^*p*.*R438H*^ variant

**Figure 6:**
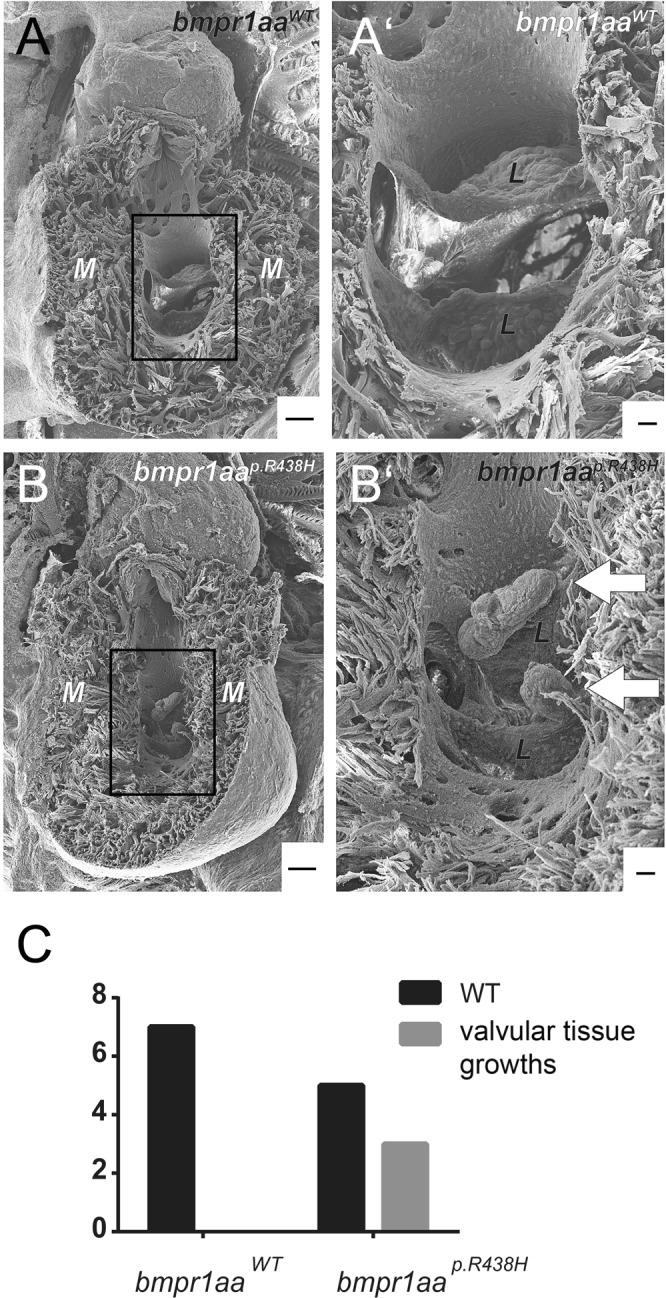
Ectopic valvular tissue growths occur in adult zebrafish overexpressing the *bmpr1aa*^*p*.*R438H*^ variant within endocardium. (**A**,**B**) Electron microscopic images of adult zebrafish hearts with (**A**) *Tg(fli1a:Gal4FF)*^*ubs3*^; *Tg(UAS:bmpr1aa*^*WT*^*_IRES_EGFP)*^*md65*^; *Tg(7xTCF-Xia*.*Sia:NLS-mCherry)*^*ia5*^ or (**B**) *Tg(fli1a:Gal4FF)*^*ubs3*^; *Tg(UAS:bmpr1aa*^*p*.*R438H*^*_IRES_EGFP)*^*md60*^; *Tg(7xTCF-Xia*.*Sia:NLS-mCherry)*^*ia5*^. M = myocardium. Scale bar = 100 µm. (**A′**,**B′**) Magnified view of the atrioventricular valve. L = valve leaflet. Scale bar = 20 µm. (**A**) Adult zebrafish hearts with endothelial overexpression of *bmpr1aa*^*WT*^ do not have any obvious morphological changes. **(B′**) 3 out of 8 analysed adult zebrafish hearts overexpressing *bmpr1aa*^*p*.*R438H*^ have ectopic valvular tissue growth at the atrioventricular valve (arrows). **(C**) Numbers of zebrafish hearts overexpressing either *bmpr1aa*^*p*.*R438H*^ or *bmpr1aa*^*WT*^ with ectopic valvular tissue growth. None out of 7 analysed zebrafish hearts overexpressing *bmpr1aa*^*WT*^ had ectopic valvular tissue growths (p = 0.200, fisher’s exact test).

To characterize AV valve morphology in adult zebrafish with an endocardial overexpression of the *bmpr1aa*^*p*.*R438H*^ variant, we performed an electron-microscopic analysis. We found that three of eight analysed hearts had some growth of ectopic tissue mass on AV valve leaflets (Fig. 6). In comparison, none of seven zebrafish hearts overexpressing *bmpr1aa*^*WT*^ displayed any such tissue growth (p = 0.200, fisher’s exact test). The ectopic tissue growths formed oblong or circular shapes in a size range of 75–150 µm in length. This finding provides further evidence for an effect of the *bmpr1a*^*p*.*R443H*^ variant on cardiac morphology.

## Discussion

As the genetics of most CHDs is unsatisfactorily explained by monogenic inheritance, more complex inheritance patterns gain importance. Here, we report a family with an exceptionally high percentage of affected members (68%, or 13 out of 19) and a potentially multigenic inheritance of CHDs. Every affected family member for which complete sequencing data is available carries both a *BMPR1A* missense mutation and a defined linkage region on chromosome 1 (Fig. 1). Therefore, an interaction of the *BMPR1A* mutation with another genetic entity within the linkage region on Chr.1 may be causative for the diseases.

BMPR1A plays a crucial role during gastrulation and differentiation of mesodermal cells^33^. As *Bmpr1a* knockout mice die by day 10 of development^44^, a complete loss of BMPR1A would presumably result in human neonatal death as well. Since embryonic development is not affected in the presented family, it appears unlikely that the *BMPR1A*^*p*.*R443H*^ mutation causes a complete loss-of-function of BMPR1A signalling. This is in agreement with our finding that the injection of human *BMPR1A*^*p*.*R443H*^ mRNA into zebrafish at the one cell stage complements the knockdown of zebrafish *bmpr1aa*. Currently, we cannot entirely exclude the possibility that the expression of human *BMPR1A*^*p*.*R443H*^ exerts a weak effect on zebrafish embryonic development. The lack of obvious embryonic or cardiac defects upon injection of *BMPR1A*^*p*.*R443H*^ mRNA into zebrafish provides further evidence for a more complex trait involving the *BMPR1A* mutation together with another modifier in causing cardiac defects. However, as family members are heterozygous carriers, the *BMPR1A*^*p*.*R443H*^ mutation may also cause a (semi-) dominant effect as assayed in transgenic zebrafish. Although continuous pan-endothelial overexpression of *bmpr1aa*^*p*.*R438H*^ does not affect embryonic valve development, adult zebrafish hearts are affected. Here, the endothelial overexpression of *bmpr1aa*^*p*.*R438H*^ leads to a clear reduction of the AV valve area when compared to the overexpression of *bmpr1aa*^*WT*^. This effect could be due to a reduced proliferation within the valve leaflets caused by lower Bmpr1aa activity.

Strikingly, adult fish overexpressing *bmpr1aa*^*p*.*R438H*^ in endocardial cells display a severe reduction of *Tg*(*7xTCF-Xla*.*Sia:NLS-mCherry*)^*ia5*^ reporter expression, which is indicative of decreased Wnt signalling activity within adult AV valves. In mice, canonical Wnt signalling acts as a mitogenic trigger of cushion mesenchyme proliferation following endoMT^42^. Indeed, injection of *apc* or *dkk-1* mRNA, both encoding inhibitors of Wnt/β-catenin signalling, induces a complete lack of embryonic endocardial cushion tissue in zebrafish. Correspondingly, zebrafish with a constitutively active Wnt/β-catenin signalling have massively expanded atrioventricular endocardial cushions^43^. Similar studies in chicken revealed an increased AVC cell number due to Wnt overexpression^45^. Several lines of evidence suggest that in mice, endoMT within the AVC is accompanied by an upregulation of Wnt/β-catenin signalling^46^. The expression of the mesenchymal cell marker αSMA is induced by TGFβ2 signalling and TGFβ2-induced endoMT depends on Wnt/β-catenin signalling^47^. Because of the known role of Wnt signalling in cardiac valve development, it is tempting to speculate that the reduced valvular Wnt signalling observed in adult *bmpr1aa*^*p*.*R438H*^*-*overexpressing zebrafish may be directly related to the reduced AV valve area. Although the exact mechanism of Wnt signalling activation within cardiac valves is currently unknown, it may be a TGF-β/Wnt cross-talk, that occurs by reciprocally-regulated ligand production, synergistically regulated shared target genes, or by cytoplasmatic protein interactions^48^. It is unclear why the reduction of the Wnt/β-catenin signalling is not apparent in the embryonic heart. Potentially, *bmpr1aa*^*p*.*R438H*^*-*overexpression may only exert a long term and weak effect on this pathway or Wnt/β-catenin signalling is more sensitive at later stages of development.

We observed that 3 out of 8 analysed adult zebrafish overexpressing *bmpr1aa*^*p*.*R438H*^ in endocardial cells develop ectopic valvular tissue mass. The occurrence of these morphological defects in the adult *bmpr1aa*^*p*.*R438H*^ mutant is not statistically significant due to the small sample size (p = 0.200, fisher’s exact test). However, the growth of ectopic valvular tissue mass indicates a tendency of the *bmpr1aa*^*p*.*R438H*^ allele to cause defective valve leaflets, something never observed among the control population of animals. Hence, the long-term endocardial overexpression of *bmpr1aa*^*p*.*R438H*^ in zebrafish did not cause the same severe cardiac defects that occur within the described family^25^. This provides additional evidence for a potentially combinatorial origin of this severe form of CHD. Similar to the human condition that is associated with the *BMPR1A*^*p*.*R443H*^ allele, continuous long-term expression of zebrafish *bmpr1aa*^*p*.*R438H*^ alone was not sufficient to cause severe morphological cardiac defects beyond size differences of the cardiac valve leaflets and growths of ectopic valvular tissue mass. This finding lends additional significance to a potentially multigenic combinatorial origin in the aetiology of these inherited CHDs. To elucidate the potential involvement of genetic modifiers within a defined genomic interval on chromosome 1 that co-segregates with the *BMPR1A*^*p*.*R443H*^ mutation in affected family members, a functional characterization of candidate genes is required.

Among the genes homologous to those present within the interval on human chromosome 1, one gene, *GIPC PDZ domain containing family*, *member 2* (*gipc2*), is a particularly strong candidate that may have a synergistic effect together with *BMPR1A*^*p*.*R443H*^ in the occurrence of CHDs due to its expression within the zebrafish heart^49^ and its interaction with TGFßR3 in regulating endoMT^50^. This TGFß receptor enhances both BMPR1A and BMPR1B signalling^51^. Such a signalling crosstalk may explain a possible genetic interaction between *GIPC2* and *BMPR1A*^*p*.*R443H*^ in the occurrence of CHDs. In preliminary own knockdown experiments in zebrafish, we have tested several candidate genes from within the 5 Mb interval, including *gipc2*, *nexn*, *eldt1*, and *fubp1*, and found that *gipc2* showed the strongest phenotype (unpublished own data). However, more substantial functional studies are required to test a potential genetic interaction between any of these candidate genes and *bmpr1aa* during cardiac valve development.

Whole genome sequencing of patient material did not reveal any coding sequence mutations in *GIPC2* or any other genes within the linkage interval on chromosome 1. Nevertheless, changes in regulatory elements or non-coding RNAs may cause alterations in gene expression that were not detected in the patient material. In addition, structural variants could be present in the linkage region, although no copy number variations (CNVs) were detected.

Taken together, we conclude that BMPR1A^p.R443H^ is a functional receptor variant that leads to a downregulation of Wnt/ß-catenin signalling, a reduced AV valve size, and ectopic valvular tissue mass in zebrafish. It is therefore a strong candidate for playing a key role in the development of the reported congenital heart defects. A complex inheritance pattern with an interaction of BMPR1A and modifiers such as GIPC2 in the aetiology of these CHDs seems plausible but needs to be functionally tested in further investigations.

## Materials and Methods

### Sequencing

SNP markers were genotyped with the “Genome-Wide Human SNP Array 6.0” by Thermo Fisher Scientific (former Affymetrix). Linkage analysis was performed using the LINKADATAGEN^52^ and MERLIN^53^ software.

Affected individual 8 was exome sequenced at the Helmholtz Zentrum Munich. Family members 13, 15, and 17 were whole-genome sequenced by Complete Genomics (Mountain View, USA) using their proprietary platform. Affected family members 4 and 22 were whole-genome sequenced by Centogene (Rostock, Germany).

We confirm that all methods were carried out in accordance with relevant guidelines and regulations. Moreover, we confirm that all experimental protocols were approved by a named institutional ethics committee (Licence-No.: Neuantrag 2/042 from March 7^th^, 2002, University of Regensburg). Informed consent was obtained from all subjects or from a parent.

### Cloning

Plasmids were generated using the Tol2kit and the Gateway system by Invitrogen^54^. Expression clones used for RNA transcription contain a CMV/SP6 promoter. Those used to generate stable transgenic lines contain a UAS Promoter. The inserted *BMPR1A* (human) ORF sequence corresponds to the Ensembl Transcript ID ENST00000372037.7. The *bmpr1aa* (zebrafish) ORF sequence corresponds to nucleotides 456 to 2039 of the Genbank sequence BC115245.1 (http://www.ncbi.nlm.nih.gov/genbank/). Mutagenesis was performed using the QuikChange II XL Site-Directed Mutagenesis Kit. Supplementary Table S3 summarizes the generated expression clones.

### Zebrafish strains and maintenance

Zebrafish were kept according to standard laboratory procedures^55^. Handling of zebrafish was done in compliance with German, Berlin and Lower-Saxony state law and carefully monitored by the local authority for animal protection [Landesamt für Gesundheit und Soziales (Berlin, Germany) and Niedersächsisches Landesamt für Verbraucherschutz und Lebensmittelsicherheit (Oldenburg, Germany)]. Breeding and harvesting of fish eggs were authorized by the State Office of consumer protection and food safety Lower-Saxony (Oldenburg, Germany) (Licence-No.: 33.19-42502-04-Demal; 06.01.2016). The generation of transgenic lines was authorized by the State Office of Health and Social Issues (LaGeSo Berlin, Germany) (Licence-No.: Reg0254/12) and by the State Office of consumer protection and food safety Lower-Saxony (Oldenburg, Germany) (Licence-No.: 33.12-42502-04-15/2012). mRNA and MO injections were performed using AB, TüLF, and WIK wildtype strains. To generate transgenic lines, AB, TüLF, and WIK wildtype strains were outcrossed with *Tg(fli1a:Gal4FF)*^*ubs3* ^^38^ or *Tg(kdrl:EGFP)*^*s843* ^^40^. These transgenic lines later were outcrossed with *Tg(7xTCF-Xla*.*Siam:nlsmCherry)*^*ia5* ^^39^.

### mRNA rescue injections

mRNA *in vitro* transcription was performed using a SP6 polymerase^56^. Zebrafish embryos were injected at 1-cell stage with 1 nl mRNA (20 ng/μl) und 1 nl morpholino oligo mix [*bmpr1aa* MO (4 mM), *bmpr1ab* MO1 (4 mM), *bmpr1ab* MO3 (4 mM)^33^ (1 mM) in Danieau’s Solution]. To prevent cell death by p53 activation due to high MO concentrations, we co-injected p53 MO (*tp53* MO4)^57^. MOs were obtained from Gene Tools and are summarized in Supplementary Table S4. Following the injections, embryos were incubated in eggwater for 24 hours and classified by the severity of their state of dorsalization^36^. Some embryos of each clutch remained uninjected and were used as negative control. Clutches with over 25% dead embryos at 24hpf in the negative control were sorted out and not analysed. Only clutches with at least n ≥ 15 (or n ≥ 10 in negative control) were analysed. The experiment was performed at least four times with each mRNA variant. The mRNA variants were blinded before injection and unblinded after dorsalization analysis.

As statistical analysis, the mean percentage of dead and C4-malformed embryos were compared between the 4 groups of MO/mRNA co-injection and a group with MO injection only. For this purpose, data was transformed using an arcsine square root transformation. Homoscedasticity and normal distribution were ensured using Levene’s test and the Shapiro-Wilk test, respectively. Data was weighted with the square of the number of embryos in the respective clutch. Afterwards the groups were compared using a two-sided student’s t-test and the data was corrected using Bonferroni-Holms procedure.

### Generation of transgenic lines

The mutation c.G1313A (p.R438H) was introduced in the zebrafish *bmpr1aa* gene and cloned into a Tol2 vector using the Gateway/Tol2 kit. Constructs were injected into 1-cell stage zebrafish embryos to generate the transgenic lines *Tg*(*UAS:bmpr1aa*^*WT*^*_IRES_EGFP*)^*md65*, *md67*^ or *Tg(UAS:bmpr1aa*^*p*.*R438H*^*_IRES_EGFP*)^*md60*,*md61*,*md66*^. The founders of these transgenic lines were used to raise stable generations, which were outcrossed with *Tg(fli1a:GAL4FF*)^*ubs3*^ and *Tg(7xTCF-Xla*.*Sia:NLS-mCherry*)^*ia5*^. As some transgenic embryos showed a mosaic endothelial expression pattern of GFP, only embryos with strong expression were selected and raised for a later inspection of AV valve morphology.

### Whole-mount *in situ* hybridization

DIG labeled probe for *egfp* was generated as previously described^58^. Whole-mount *in situ* hybridization experiments were performed as previously described^59^. Images were recorded on a stereomicroscope (Leica M165 FC) with an EOS 5 D Mark III (Canon) camera and processed using Adobe Illustrator (Adobe Systems).

### PTU treatment and fixation

*Tg(fli1a:GAL4FF*)^*ubs3*^; *Tg(UAS:bmpr1aa*^*WT*^*_IRES_EGFP)*/*Tg(UAS:bmpr1aa*^*p*.*R438H*^*_IRES_EGFP)*; *Tg(7xTCF-Xla*.*Sia:NLS-mCherry)*^*ia5*^ embryos were treated with PTU (Sigma) at 24hpf, anaesthetized with 3-aminobenzoic acid ethyl ester (Tricaine) (Sigma) and fixed in 4% PFA at 120dpf.

### Adult heart extraction

Adult heart extraction was performed according to the published protocol^60^. After extraction of the heart, the atrium was carefully severed from the ventricle exposing the AV valve. Hearts were embedded in 1% low-melting agarose in glass-bottom dishes with the AV valve facing down and imaged.

### Immunohistochemistry

Whole-mount antibody stainings of zebrafish embryos were performed as previously described^61^. The following antibodies were used: mouse anti–ALCAM/Dm-GRASP/Neurolin (1:200; Developmental Studies Hybridoma Bank). Nuclear stainings were performed using 4′,6-diamidino-2-phenylindole (DAPI) (Sigma). We used secondary antibodies conjugated to Alexa-561 or Alexa-647 (Life Technologies) at 1:200.

### Preparation and fixation of adult hearts for scanning electron microscopy (SEM)

Adult fishes were anaesthesized and transferred to a Petri dish filled with cool Locke’s solution (6 °C). The bottom of the Petri dish was covered with a layer of wax. Insect needles were inserted into the mouths, caudal trunks and pectoral fins of the fishes to physically fix them to the bottom of the Petri dish. The pericardial cavity was then opened using microsurgical scissors and the still beating hearts were perfused with Locke’s solution (via a micropipette inserted into the sinus venosus) until all visible signs of blood were removed from the heart and bulbus arteriosus. To fix hearts in a general dilation, final perfusion was carried out with a calcium-free Locke’s solution of 20 mmol/l manganese chloride^62^. MnCl_2_ causes a cardiac arrest in a general dilation by calcium channel blocking. After cardiac arrest, the hearts were externally rinsed with a 25% solution of glutaraldehyde to achieve a rapid pre-fixation of specimens^63^. Final fixation of the specimens was carried out in a 2% solution of glutaraldehyde followed by post-fixation in Bouin’s solution according to established protocols^64^. The fixed specimens were dehydrated in the usual manner and dried by the critical point method. The dried specimens were mounted on aluminum taps with conducting silver and their ventricles were opened by removal of their ventral myocardial walls using electrolytically sharpened tungsten needles. Specimens were sputter-coated with platinum-palladium (Leica EM ACE 200).

### Image acquisition

Confocal images were obtained using a Leica TCS SP8 confocal laser microscope with 20x and 40x magnification for adult and embryonic heart valves, respectively. 3D projections and oblique slices of confocal images were generated using Imaris (Bitplane). Electron microscopy (SEM) images were obtained using a Zeiss Ultra *plus* field emission scanning electron microscope. Images were processed using Adobe Illustrator CC2015 (Adobe Systems).

### Quantification of valve parameters and statistical analysis

Diameter and area of AV valves/AVCs were measured using Fiji^65^. Embryonic AVC diameter was compared using a two-sided student’s t-test. Homoscedasticity and normal distribution were ensured using Levene’s test and the Shapiro-Wilk test, respectively. Standard deviation is reported as measure of variability. Adult valve sizes were determined by the measurement of the area bounded by the AV valve annulus. This valve annulus surrounds the edge of the valve leaflets marked by TCF expression and occurs in the confocal images as a black ring. The AV valve measurement technique is shown in detail in Supplementary Fig. S4. The individual measurement data is shown in Supplementary Table S13. For statistical comparison of this adult valve area, analysis of covariance (ANCOVA) was used, which uses linear regression to statistically remove the effects of fish length as covariate before performing a standard ANOVA. Means of AV valve area are reported adjusted for the covariate zebrafish length using ANCOVA.

## Supplementary information

Supplementary Information

## Data Availability

Data generated or analysed during zebrafish experiments are included in this published article (and its Supplementary Information files). Sequencing data and all microscopic images are available from the corresponding authors on reasonable request.
