# Supplementary material for: A familial congenital heart disease with a possible multigenic origin involving a mutation in BMPR1A
Source: Sci Rep. 2019 Feb 27;9:2959. doi: 10.1038/s41598-019-39648-7 (PMC6393482; doi:10.1038/s41598-019-39648-7)
Supplement: Supplementary file 1 — Supplementary Information [file 41598_2019_39648_MOESM1_ESM.docx]

**Supplementary material to**

**A familial congenital heart disease with a possible multigenic origin involving a mutation in BMPR1A**

Till Joscha Demal^1,2§^; Melina Heise^3§^; Benedikt Reiz^1^; Deepika Dogra^4^, Ingrid Brænne^1,5^; Hermann Reichenspurner^2^; Jörg Männer^6^, Zouhair Aherrahrou^1^; Heribert Schunkert^7^; Jeanette Erdmann^1*^; Salim Abdelilah-Seyfried^3,4*^

^§^ both authors contributed equally

1 Institute for Cardiogenetics, University Heart Centre Lübeck, University of Lübeck, DZHK (German Research Centre for Cardiovascular Research) partner site Hamburg/Lübeck/Kiel, D-23562 Lübeck, Germany

2 Department of Cardiovascular Surgery, University Heart Centre Hamburg, D-20246 Hamburg, Germany

3 Institute of Molecular Biology, Hannover Medical School, D-30625 Hannover, Germany

4 Institute of Biochemistry and Biology, Potsdam University, D-14476 Potsdam, Germany

5 Center for Public Health Genomics, University of Virginia, Charlottesville, VA 22903 USA

6 Institute of Anatomy and Embryology, UMG, Göttingen University, D−37075 Göttingen, Germany

7 Department of Cardiovascular Diseases, German Heart Centre Munich, Technical University of Munich (TUM) and DZHK (German Research Centre for Cardiovascular Research) partner site, D-80636 Munich, Germany

| **ID** | **Ebstein's Anomaly** | **Wolff-Parkinson-White syndrome** | **Septal Defects** | **Cleft Mitral Valve** | **Right Bundle Branch Block** | **Other /**  **Unknown** |
| --- | --- | --- | --- | --- | --- | --- |
| 2 | - | - | - | - | - | X |
| 3 | - | - | - | - | - | X |
| 4 | X | - | X | X | - | - |
| 5 | - | - | - | - | - | X |
| 7 | X | X | X | X | - | - |
| 8 | - | - | - | - | - | X |
| 9 | - | - | - | - | - | X |
| 10 | X | X | - | X | - | - |
| 14 | - | - | X | - | X | - |
| 15 | X | X | X | - | X | - |
| 17 | - | - | X | - | X | - |
| 22 | - | - | - | - | - | X |
| 25 | - | - | - | - | - | X |

Table S1: Phenotypes of affected family members.

*This table lists the main phenotypes found in the described family. All features are based on the publication by Schunkert et al.*^1^ *or reports by family members. For some individuals, no specific diagnosis is available and not all presumably affected family members underwent clinical examination, for several reasons. Septal defects include AVSDs, ASDs, and VSDs. Fig. 1 shows a pedigree of the described family. Detailed information on each patient are presented in the following:*

**1st Generation**

*Patient 2: Father of the index patient (patient 7). He died due to cardiac disease without known specific diagnosis.*

**2nd Generation**

*Patient 3: Sister of the index patient. She was diagnosed with a bicuspid and stenotic aortic valve.*

*Patient 4: Sister of the index patient. She was diagnosed with Ebstein’s anomaly, cleft anterior mitral leaflet, and ASD.*

*Patient 5: Brother of the index patient. He died a few hours after birth without specific diagnosis. His mother reported, that he suffered from “severe malformations”.*

*Patient 7: This is the index patient, which was diagnosed with Ebstein’s anomaly, cleft anterior mitral leaflet with moderate mitral regurgitation, WPW syndrome, and episodes of a focal atrial tachycardia. In addition, an AVSD was partially corrected at the age of 7.*

*Patient 8: Sister of the index patient. She appeared clinically healthy, but presented with a prominent systolic ejection murmur in projection to the left ventricular outflow tract. Subsequent examination revealed a membraneous aneurysm in the left ventricular outflow tract that bowed into the right ventricle. However, no ventricular septum defect was detected.*

*Patient 9: Sister of the index patient. She died three days after birth. Her mother reported, that she suffered from a heart defect.*

**3rd Generation**

*Patient 10: Daughter of Patient 3. She suffered from Ebstein’s anomaly, a cleft mitral leaflet with moderate regurgitation, and a Wolff-Parkinson-White syndrome.*

*Patient 14: Daughter of the index patient. She was diagnosed with a combination of an AVSD and a complete right bundle branch block.*

*Patient 15: Son of the index patient. He was diagnosed with Ebstein’s anomaly, a small VSD, a complete right bundle branch block, and a Wolff-Parkinson-White syndrome.*

*Patient 17: Daughter of patient 8. She was diagnosed with an AVSD and a complete right bundle branch block.*

**4th Generation**

*Patient 22: Daughter of Patient 10. She was not part of the original publication. Referring to reports of her family, she is affected by some cardiac malformation (no medical records available).*

*Patient 25: Daughter of patient 14. She was not part of the original publication. Referring to reports of her family, she is affected by some cardiac malformation (no medical records available).*

*In addition, the original publication reports that none of 7 aunts, 5 uncles, and 45 nieces and nephews of the index patient were diagnosed with any CHD. However, more family members might be affected to some degree.*

*
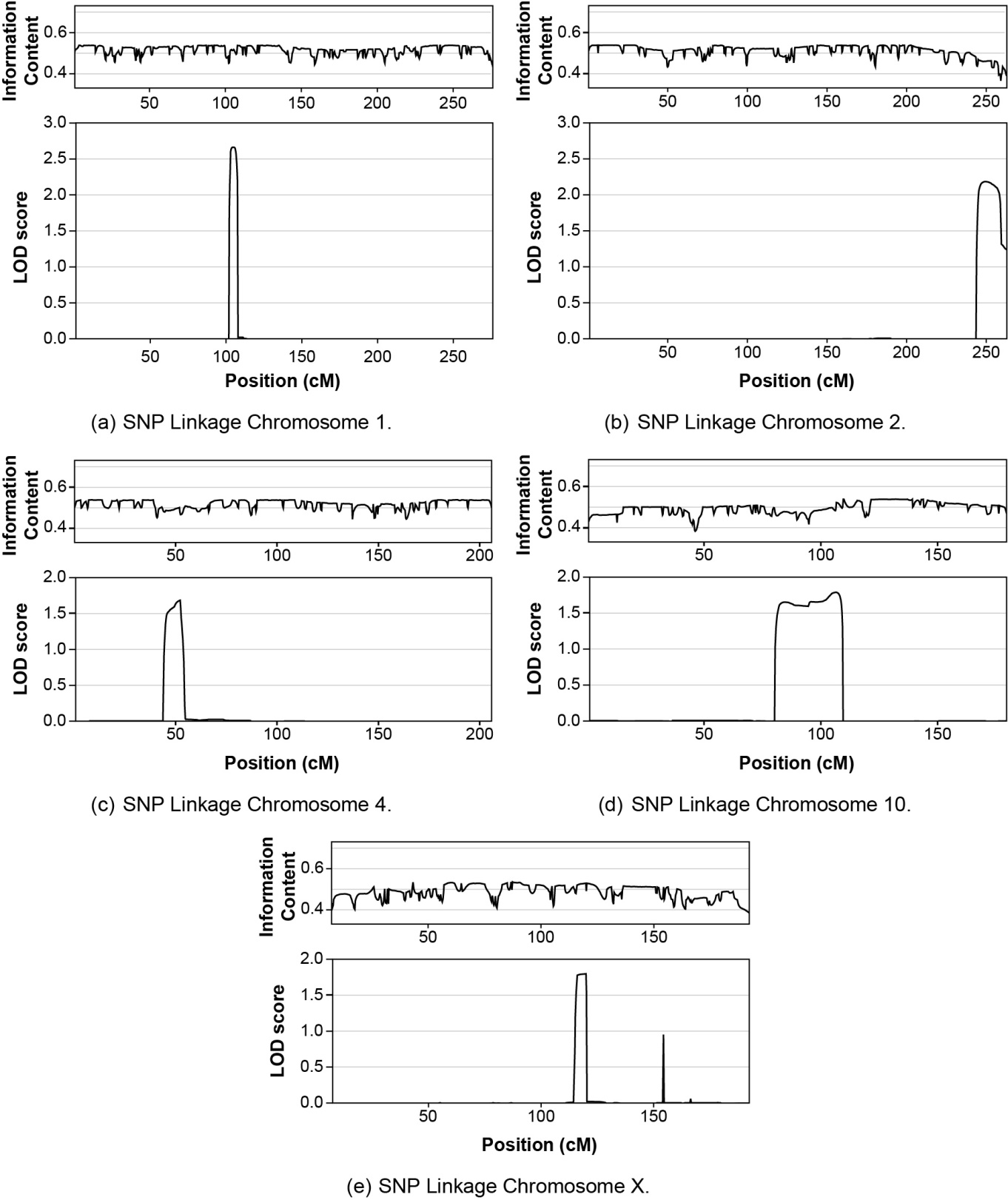
*

Fig. S1: Nonparametric linkage results for all chromosomes with LOD ≥ 1.5. The linkage data shown is based on SNP data from the Genome-Wide Human SNP Array 6.0 (Affymetrix) and calculated with MERLIN using the exponential model by Kong and Cox^2^. The maximal LOD scores are: Chromosome 1 = 2.663; Chromosome 2 = 2.183; Chromosome 4 = 1.683; Chromosome 10 = 1.776; Chromosome X = 1.793.

| **BMPR1A variant**  **(human)** | **Bmpr1aa orthologue (zebrafish)** | **Associated Phenotype** | **Source** |
| --- | --- | --- | --- |
| p.R443H | p.R438H | Ebstein’s anomaly, atrioventricular septal defect, aortic stenosis, and others | Whole-Exome-Sequencing of the family reported by Schunkert et al., 1997^1^ |
| p.R443C | p.R438C | Juvenile polyposis syndrome (JPS) | Howe et al., 2004^3^ |
| p.L342R  (“Linkspoot“) | p.L337R  (“Linkspoot“) | Defects of dorsoventral patterning and left-right axis specification in zebrafish | Smith et al., 2011^4^ |

Table S2: Studied BMPR1A variants


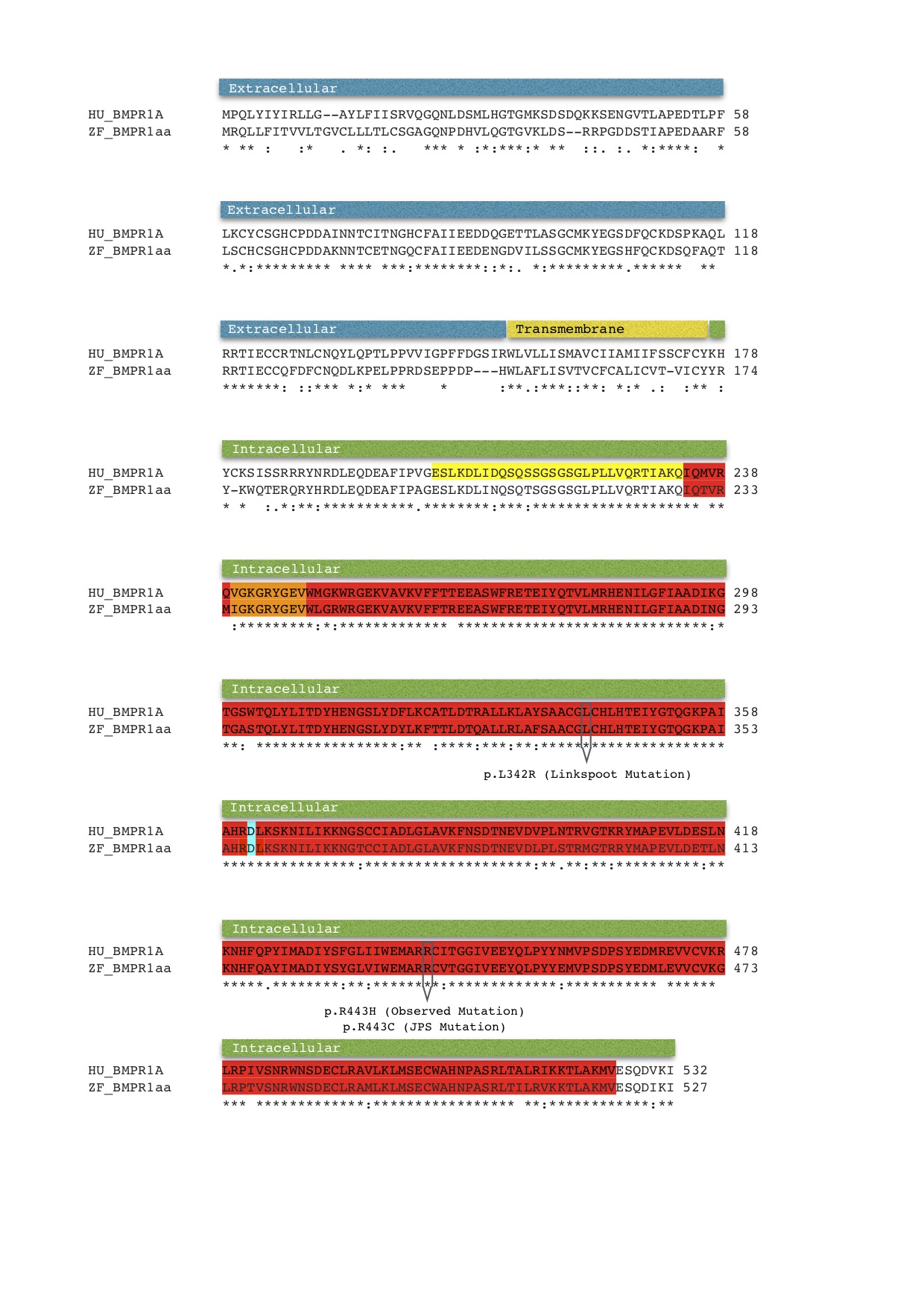


***Fig. S2:*** Sequence alignment of the human BMPR1A (HU_BMPR1A) and the zebrafish homologue Bmpr1aa (ZF_BMPR1aa) according to ClustalW2 (https://www.ebi.ac.uk/Tools/msa/clustalw2/). The sequence annotations are labelled according to Uniprot (https://www.uniprot.org) and Prosite (https://prosite.expasy.org). The protein kinase domain is labelled in red, the ATP-binding site is labelled in orange, the active site (Proton Acceptor) is labelled in blue and the GS domain is labelled in yellow. Asterisks indicate positions which have a single, fully conserved residue. Colons indicare conservation between the homologues of strongly similar properties (scoring >0.5 in the Gonnet PAM 250 matrix). Periods indicate conservations between the homologues of weakly similar properties (scoring ≤0.5 in the Gonnet PAM 250 matrix).

| **Destination vector** | **p5E-Entryclone** | **Middle Entryclone** | **Mutation** | **p3E-Entryclone** |
| --- | --- | --- | --- | --- |
| pDest Tol2pA | p5E-CMV/SP6 | *BMPR1A* (human) |  | p3E-IRES-EGFPpA |
| “ | “ | *BMPR1A* (human) | c.G1328A (p.R443H) | “ |
| “ | “ | *BMPR1A* (human) | c.C1327T (p.R443C) | “ |
| “ | “ | *BMPR1A* (human) | c.T1025G (p.L342R) | “ |
| “ | p5E-UAS | *bmpr1aa* (zebrafish) |  | “ |
| “ | “ | *bmpr1aa* (zebrafish) | c.G1313A (p.R438H) | “ |

Table S3: Generated expression clones

| **Morpholino Name** | **Target Gene** | **Sequence (5’ – 3’)** |
| --- | --- | --- |
| *bmpr1aa* MO | *bmpr1aa* (*alk3a*) | GACGCATTGTCAAATTGTCTTGTCG |
| *bmpr1ab* MO1 | *bmpr1ab* (*alk3b*) | GTCGAGTTGTTGAACTGTATGGCTG |
| *bmpr1ab* MO3 | *bmpr1ab* (*alk3b*) | TGAAGATGAATGGACACGGTAAGAG |
| *tp53* MO4 | *tp53* | GCGCCATTGCTTTGCAAGAATTG |

Table S4: Morpholino Oligos used in the rescue studies

| **Target** | **Sequence (5’ – 3’)** |
| --- | --- |
| *gipc2*, ATGMO | ACACGGCAAACAGACAGCCAGAGCA |
| *gipc2*, spliceMO; exon1/intron1 splice-donor site | TTATTTGGCATTTACCTCATCTGGA |

Table S5: Morpholino oligos used for the knockdown of gipc2

**
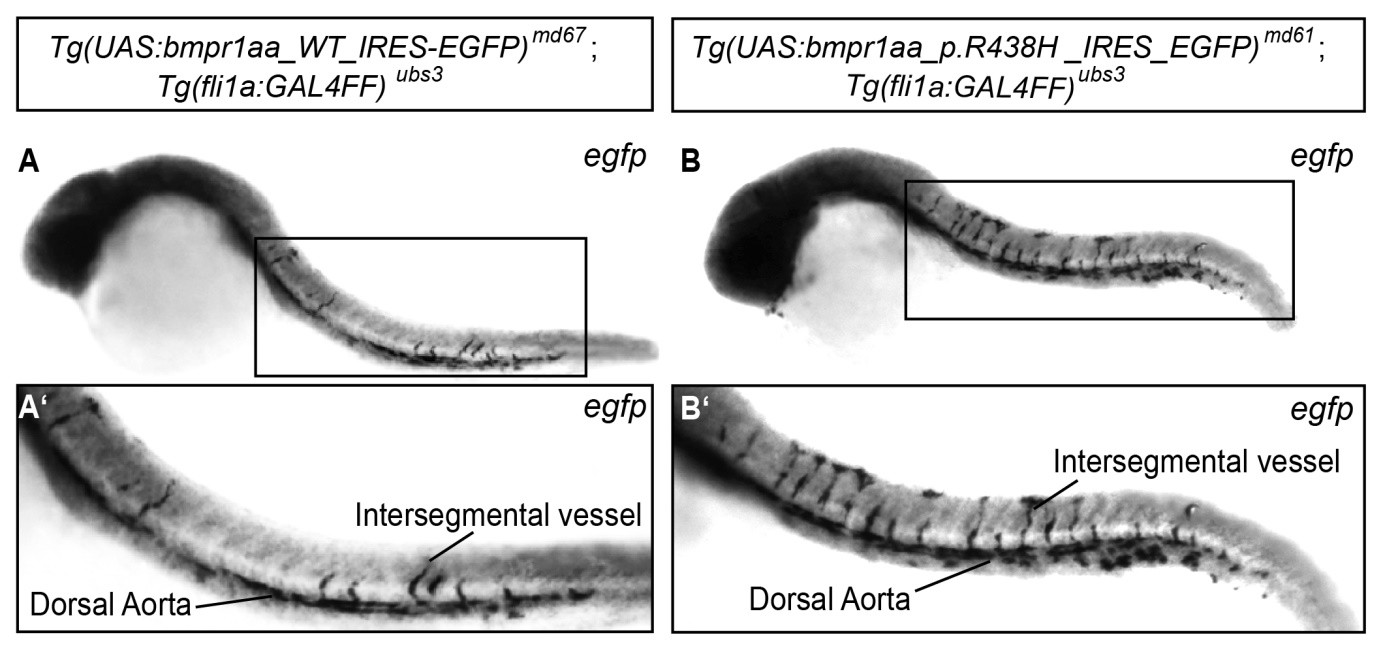
**

Fig. S3: Validation of endothelial-specific bmpr1aa transgenic overexpression. (A, A’) Expression analysis of egfp by whole-mount in situ hybridization in Tg(UAS:bmpr1aa^WT^_IRES_EGFP)^md67^; Tg(fli1a:GAL4FF)^ubs3^ embryos at 30 hpf. Mosaic egfp expression is detected in Dorsal aorta (DA) and Intersegmental vessels (ISVs). (B,B’) Expression analysis of egfp by whole-mount in situ hybridization in Tg(UAS:bmpr1aa^p.R438H^_IRES_EGFP)^md61^; Tg(Fli1a:GAL4FF)^ubs3^ embryos at 30 hpf. Mosaic egfp expression is detected in DA and ISVs.


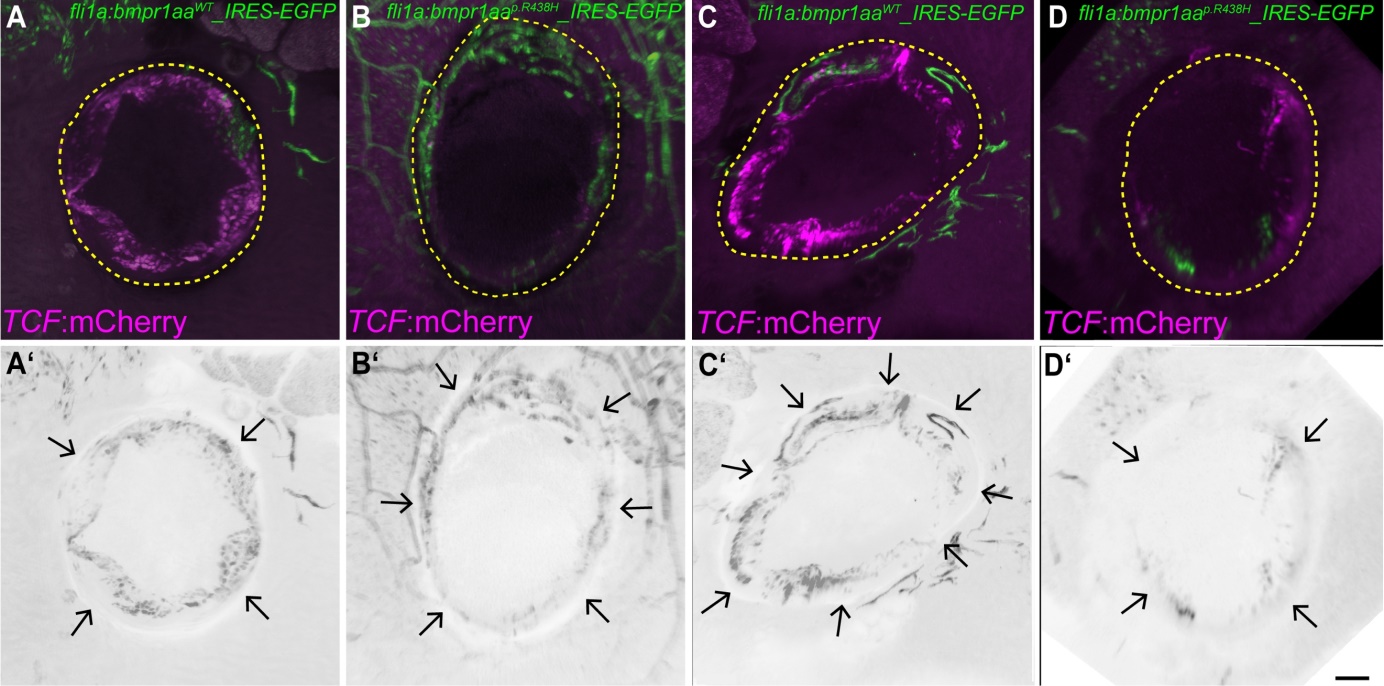


Fig. S4: Adult zebrafish with pan-endothelial expression of Bmpr1aa^WT^ or Bmpr1aa^p.R438H^. Confocal z-stack maximum intensity projection of adult AV valves of zebrafish with (A(‘), C(‘)) Tg(fli1a:Gal4FF)^ubs3^; Tg(UAS:bmpr1aa^WT^_IRES_EGFP)^md65^; Tg(7xTCF-Xia.Sia:NLS-mCherry)^ia5^ or (B(‘), D(‘)) Tg(fli1a:Gal4FF)^ubs3^; Tg(UAS:bmpr1aa^p.R438H^_IRES_EGFP)^md60^; Tg(7xTCF-Xia.Sia:NLS-mCherry)^ia5^. Fish overexpressing bmpr1aa^p.R438H^ within endocardium display a severe reduction of Tg(7xTCF-Xla.Sia:NLS-mCherry)^ia5^ reporter expression in comparison to fish overexpressing bmpr1aa^WT^. Adult AV valve measurement technique: The interrupted line indicates the edge of the valve leaflets marked by TCF expression (A-D). The interrupted line in A-D lies directly on the AV valve annulus indicated by arrows in A’-D’. The area bounded by the AV valve annulus was measured to quantify the valve size. Scale bar = 50µm.

**EXPERIMENTAL DATA**

mRNA complementation assay

|  | **n** | **WT** | **C1** | **C2** | **C3** | **C4** | **C5 (dead)** | **other** |
| --- | --- | --- | --- | --- | --- | --- | --- | --- |
| Clutch 1 | 35 | 34 (97.1%) | 1 (2.9%) | 0 (0.0%) | 0 (0.0%) | 0 (0.0%) | 0 (0.0%) | 0 (0.0%) |
| Clutch 2 | 13 | 12 (92.3%) | 0 (0.0%) | 0 (0.0%) | 0 (0.0%) | 0 (0.0%) | 1 (7.7%) | 0 (0.0%) |
| Clutch 3 | 14 | 12 (85.7%) | 0 (0.0%) | 0 (0.0%) | 0 (0.0%) | 0 (0.0%) | 2 (14.3%) | 0 (0.0%) |
| Clutch 4 | 33 | 33 (100.0%) | 0 (0.0%) | 0 (0.0%) | 0 (0.0%) | 0 (0.0%) | 0 (0.0%) | 0 (0.0%) |
| Clutch 5 | 32 | 31 (96.9%) | 0 (0.0%) | 0 (0.0%) | 0 (0.0%) | 0 (0.0%) | 1 (3.1%) | 0 (0.0%) |
| Clutch 6 | 26 | 22 (84.6%) | 0 (0.0%) | 0 (0.0%) | 0 (0.0%) | 0 (0.0%) | 4 (15.4%) | 0 (0.0%) |
| Clutch 7 | 23 | 23 (100.0%) | 0 (0.0%) | 0 (0.0%) | 0 (0.0%) | 0 (0.0%) | 0 (0.0%) | 0 (0.0%) |
| Clutch 8 | 18 | 18 (100.0%) | 0 (0.0%) | 0 (0.0%) | 0 (0.0%) | 0 (0.0%) | 0 (0.0%) | 0 (0.0%) |
| Clutch 9 | 13 | 13 (100.0%) | 0 (0.0%) | 0 (0.0%) | 0 (0.0%) | 0 (0.0%) | 0 (0.0%) | 0 (0.0%) |
| Clutch 10 | 14 | 14 (100.0%) | 0 (0.0%) | 0 (0.0%) | 0 (0.0%) | 0 (0.0%) | 0 (0.0%) | 0 (0.0%) |
| Clutch 11 | 30 | 30 (100.0%) | 0 (0.0%) | 0 (0.0%) | 0 (0.0%) | 0 (0.0%) | 0 (0.0%) | 0 (0.0%) |
| Clutch 12 | 38 | 38 (100.0%) | 0 (0.0%) | 0 (0.0%) | 0 (0.0%) | 0 (0.0%) | 0 (0.0%) | 0 (0.0%) |
| Clutch 13 | 34 | 32 (94.1%) | 0 (0.0%) | 0 (0.0%) | 0 (0.0%) | 0 (0.0%) | 1 (2.9%) | 1 (2.9%) |
| Clutch 14 | 17 | 14 (82.4%) | 1 (5.9%) | 0 (0.0%) | 0 (0.0%) | 0 (0.0%) | 2 (11.8%) | 0 (0.0%) |
| Clutch 15 | 26 | 25 (96.2%) | 0 (0.0%) | 0 (0.0%) | 0 (0.0%) | 0 (0.0%) | 1 (3.8%) | 0 (0.0%) |
| Clutch 16 | 51 | 48 (94.1%) | 1 (2.0%) | 0 (0.0%) | 0 (0.0%) | 0 (0.0%) | 2 (3.9%) | 0 (0.0%) |
| Clutch 17 | 12 | 12 (100.0%) | 0 (0.0%) | 0 (0.0%) | 0 (0.0%) | 0 (0.0%) | 0 (0.0%) | 0 (0.0%) |
| Clutch 18 | 14 | 14 (100.0%) | 0 (0.0%) | 0 (0.0%) | 0 (0.0%) | 0 (0.0%) | 0 (0.0%) | 0 (0.0%) |
| Clutch 19 | 25 | 23 (92.0%) | 0 (0.0%) | 0 (0.0%) | 0 (0.0%) | 0 (0.0%) | 2 (8.0%) | 0 (0.0%) |
| Clutch 20 | 21 | 17 (81.0%) | 1 (4.8%) | 0 (0.0%) | 0 (0.0%) | 0 (0.0%) | 2 (9.5%) | 1 (4.8%) |
| Clutch 21 | 28 | 21 (75.0%) | 0 (0.0%) | 0 (0.0%) | 0 (0.0%) | 0 (0.0%) | 6 (21.4%) | 1 (3.6%) |
| Clutch 22 | 11 | 10 (90.9%) | 1 (9.1%) | 0 (0.0%) | 0 (0.0%) | 0 (0.0%) | 0 (0.0%) | 0 (0.0%) |
| **TOTAL** | 528 | 496 | 5 | 0 | 0 | 0 | 24 | 3 |
| **MEAN** | 24.0 | 22.5 (**93.7%**) | 0.2 (**1.1%**) | 0.0 (**0.0%**) | 0.0 (**0.0%**) | 0.0 (**0.0%**) | 1.1 (**4.6%**) | 0.1 (**0.5%**) |

Table S6: Classes of dorsalization phenotypes in the group without injection of MOs or mRNA (uninjected) 24hpf.

|  | **n** | **WT** | **C1** | **C2** | **C3** | **C4** | **C5 (dead)** | **other** |
| --- | --- | --- | --- | --- | --- | --- | --- | --- |
| Clutch 1 | 55 | 0 (0.0%) | 0 (0.0%) | 0 (0.0%) | 4 (7.3%) | 2 (3.6%) | 49 (89.1%) | 0 (0.0%) |
| Clutch 2 | 42 | 0 (0.0%) | 0 (0.0%) | 0 (0.0%) | 3 (7.1%) | 5 (11.9%) | 34 (81.0%) | 0 (0.0%) |
| Clutch 3 | 49 | 5 (10.2%) | 1 (2.0%) | 1 (2.0%) | 3 (6.1%) | 3 (6.1%) | 36 (73.5%) | 0 (0.0%) |
| Clutch 4 | 47 | 0 (0.0%) | 0 (0.0%) | 0 (0.0%) | 0 (0.0%) | 3 (6.4%) | 44 (93.6%) | 0 (0.0%) |
| Clutch 5 | 24 | 0 (0.0%) | 0 (0.0%) | 0 (0.0%) | 0 (0.0%) | 0 (0.0%) | 24 (100.0%) | 0 (0.0%) |
| **TOTAL** | 217 | 5 | 1 | 1 | 10 | 13 | 187 | 0 |
| **MEAN** | 43.4 | 1.0 (**2.0%**) | 0.2 (**0.4%**) | 0.2 (**0.4%**) | 2.0 (**4.1%**) | 2.6 (**5.6%**) | 37.4 (**87.4%**) | 0.0 (**0.0%**) |

Table S7: Classes of dorsalization phenotypes in the group with injection of bmpr1aa/ab MOs only (knockdown) 24hpf.

|  | **n** | **WT** | **C1** | **C2** | **C3** | **C4** | **C5 (dead)** | **other** |
| --- | --- | --- | --- | --- | --- | --- | --- | --- |
| Clutch 1 | 17 | 1 (5.9%) | 1 (5.9%) | 4 (23.5%) | 4 (23.5%) | 1 (5.9%) | 6 (35.3%) | 0 (0.0%) |
| Clutch 2 | 40 | 3 (7.5%) | 15 (37.5%) | 6 (15.0%) | 11 (27.5%) | 0 (0.0%) | 5 (12.5%) | 0 (0.0%) |
| Clutch 3 | 44 | 0 (0.0%) | 2 (4.5%) | 4 (9.1%) | 18 (40.9%) | 5 (11.4%) | 15 (34.1%) | 0 (0.0%) |
| Clutch 4 | 31 | 2 (6.5%) | 6 (19.4%) | 1 (3.2%) | 8 (25.8%) | 1 (3.2%) | 13 (41.9%) | 0 (0.0%) |
| Clutch 5 | 58 | 6 (10.3%) | 9 (15.5%) | 3 (5.2%) | 25 (43.1%) | 3 (5.2%) | 12 (20.7%) | 0 (0.0%) |
| Clutch 6 | 38 | 0 (0.0%) | 0 (0.0%) | 0 (0.0%) | 11 (28.9%) | 1 (2.6%) | 26 (68.4%) | 0 (0.0%) |
| **TOTAL** | 228 | 12 | 33 | 18 | 77 | 11 | 77 | 0 |
| **MEAN** | 38.0 | 2.0 (**5.0%**) | 5.5 (**13.8%**) | 3.0 (**9.3%**) | 12.8 (**31.6%**) | 1.8 (**4.7%**) | 12.8 (**35.5%**) | 0.0 (**0.0%**) |

Table S8: Classes of dorsalization phenotypes in the group with co-injection of bmpr1aa/ab MOs and BMPR1A^WT^ mRNA 24hpf.

|  | **n** | **WT** | **C1** | **C2** | **C3** | **C4** | **C5 (dead)** | **other** |
| --- | --- | --- | --- | --- | --- | --- | --- | --- |
| Clutch 1 | 48 | 9 (18.8%) | 2 (4.2%) | 2 (4.2%) | 9 (18.8%) | 4 (8.3%) | 22 (45.8%) | 0 (0.0%) |
| Clutch 2 | 30 | 1 (3.3%) | 8 (26.7%) | 5 (16.7%) | 14 (46.7%) | 0 (0.0%) | 2 (6.7%) | 0 (0.0%) |
| Clutch 3 | 66 | 0 (0.0%) | 4 (6.1%) | 5 (7.6%) | 32 (48.5%) | 7 (10.6%) | 18 (27.3%) | 0 (0.0%) |
| Clutch 4 | 48 | 2 (4.2%) | 0 (0.0%) | 0 (0.0%) | 26 (54.2%) | 2 (4.2%) | 18 (37.5%) | 0 (0.0%) |
| Clutch 5 | 63 | 1 (1.6%) | 0 (0.0%) | 0 (0.0%) | 6 (9.5%) | 4 (6.3%) | 52 (82.5%) | 0 (0.0%) |
| **TOTAL** | 255 | 13 | 14 | 12 | 87 | 17 | 112 | 0 |
| **MEAN** | 51.0 | 2.6 (**5.6%**) | 2.8 (**7.4%**) | 2.4 (**5.7%**) | 17.4 (**35.5%**) | 3.4 (**5.9%**) | 22.4 (**40.0%**) | 0.0 (**0.0%**) |

Table S9: Classes of dorsalization phenotypes in the group with co-injection of bmpr1aa/ab MOs and BMPR1A^p.R443H^ mRNA 24hpf.

|  | **n** | **WT** | **C1** | **C2** | **C3** | **C4** | **C5 (dead)** | **other** |
| --- | --- | --- | --- | --- | --- | --- | --- | --- |
| Clutch 1 | 21 | 1 (4.8%) | 1 (4.8%) | 1 (4.8%) | 6 (28.6%) | 2 (9.5%) | 10 (47.6%) | 0 (0.0%) |
| Clutch 2 | 51 | 0 (0.0%) | 0 (0.0%) | 3 (5.9%) | 17 (33.3%) | 1 (2.0%) | 30 (58.8%) | 0 (0.0%) |
| Clutch 3 | 53 | 0 (0.0%) | 2 (3.8%) | 1 (1.9%) | 14 (26.4%) | 4 (7.5%) | 32 (60.4%) | 0 (0.0%) |
| Clutch 4 | 22 | 2 (9.1%) | 0 (0.0%) | 0 (0.0%) | 8 (36.4%) | 2 (9.1%) | 10 (45.5%) | 0 (0.0%) |
| **TOTAL** | 147 | 3 | 3 | 5 | 45 | 9 | 82 | 0 |
| **MEAN** | 36.8 | 0.8 (**3.5%**) | 0.8 (**2.1%**) | 1.3 (**3.1%**) | 11.3 (**31.2%**) | 2.3 (**7.0%**) | 20.5 (**53.1%**) | 0.0 (**0.0%**) |

Table S10: Classes of dorsalization phenotypes in the group with co-injection of bmpr1aa/ab MOs and BMPR1A^p.R443C^ mRNA (JPS) 24hpf.

|  | **n** | **WT** | **C1** | **C2** | **C3** | **C4** | **C5 (dead)** | **other** |
| --- | --- | --- | --- | --- | --- | --- | --- | --- |
| Clutch 1 | 47 | 0 (0.0%) | 0 (0.0%) | 1 (2.1%) | 0 (0.0%) | 1 (2.1%) | 44 (93.6%) | 1 (2.1%) |
| Clutch 2 | 49 | 0 (0.0%) | 0 (0.0%) | 1 (2.0%) | 0 (0.0%) | 1 (2.0%) | 47 (95.9%) | 0 (0.0%) |
| Clutch 3 | 52 | 3 (5.8%) | 1 (1.9%) | 1 (1.9%) | 1 (1.9%) | 1 (1.9%) | 45 (86.5%) | 0 (0.0%) |
| Clutch 4 | 71 | 1 (1.4%) | 0 (0.0%) | 1 (1.4%) | 0 (0.0%) | 1 (1.4%) | 68 (95.8%) | 0 (0.0%) |
| **TOTAL** | 219 | 4 | 1 | 4 | 1 | 4 | 204 | 1 |
| **MEAN** | 54.8 | 1.0 (**1.8%**) | 0.3 (**0.5%**) | 1.0 (**1.9%**) | 0.3 (**0.5%**) | 1.0 (**1.9%**) | 51.0 (**93.0%**) | 0.3 (**0.5%**) |

Table S11: Classes of dorsalization phenotypes in the group with co-injection of bmpr1aa/ab MOs and BMPR1A^p.L342R^ mRNA (Linkspoot) 24hpf.

AVC / AV valve measurements in transgenic zebrafish

| **No.** | **Genotype** | **AVC diameter (μm)** |
| --- | --- | --- |
| 1 | bmpr1aa^WT^ | 85.6 |
| 2 | bmpr1aa^WT^ | 67.6 |
| 3 | bmpr1aa^WT^ | 83.6 |
| 4 | bmpr1aa^WT^ | 69.2 |
| 5 | bmpr1aa^WT^ | 72.6 |
| 6 | bmpr1aa^WT^ | 66.0 |
| 7 | bmpr1aa^p.R438H^ | 29.8 |
| 8 | bmpr1aa^p.R438H^ | 79.8 |
| 9 | bmpr1aa^p.R438H^ | 63.4 |
| 10 | bmpr1aa^p.R438H^ | 59.4 |
| 11 | bmpr1aa^p.R438H^ | 69.3 |
| 12 | bmpr1aa^p.R438H^ | 52.7 |
| 13 | bmpr1aa^p.R438H^ | 55.8 |
| 14 | bmpr1aa^p.R438H^ | 71.0 |

Table S12: Measurements of AVC diameter (μm) in Tg(fli1a:Gal4FF)^ubs3^; Tg(UAS:bmpr1aa_IRES_EGFP); Tg(7xTCF-Xia.Sia:NLS-mCherry)^ia5^ embryos 120hpf overexpressing either bmpr1aa^WT^ or bmpr1aa^p.R438H^ within endocardium/endothelium.

| **No.** | **Genotype** | **AV valve area (um^2^)** | **Fish length (mm)** |
| --- | --- | --- | --- |
| 1 | bmpr1aa^WT^ | 192,950 | 27 |
| 2 | bmpr1aa^WT^ | 155,417 | 28 |
| 3 | bmpr1aa^WT^ | 151,484 | 27 |
| 4 | bmpr1aa^WT^ | 77,490 | 20 |
| 5 | bmpr1aa^WT^ | 121,873 | 21 |
| 6 | bmpr1aa^WT^ | 83,220 | 24 |
| 7 | bmpr1aa^WT^ | 132,926 | 22 |
| 8 | bmpr1aa^WT^ | 82,941 | 21 |
| 9 | bmpr1aa^WT^ | 84,877 | 20 |
| 10 | bmpr1aa^WT^ | 109,570 | 30 |
| 11 | bmpr1aa^WT^ | 158,275 | 25.5 |
| 12 | bmpr1aa^WT^ | 183,666 | 25 |
| 13 | bmpr1aa^p.R438H^ | 67,119 | 27 |
| 14 | bmpr1aa^p.R438H^ | 100,536 | 26.5 |
| 15 | bmpr1aa^p.R438H^ | 64,278 | 26 |
| 16 | bmpr1aa^p.R438H^ | 139,896 | 28 |
| 17 | bmpr1aa^p.R438H^ | 99,954 | 26 |
| 18 | bmpr1aa^p.R438H^ | 151,352 | 28 |
| 19 | bmpr1aa^p.R438H^ | 102,199 | 29 |
| 20 | bmpr1aa^p.R438H^ | 125,707 | 27 |
| 21 | bmpr1aa^p.R438H^ | 79,387 | 23 |
| 22 | bmpr1aa^p.R438H^ | 96,531 | 26 |
| 23 | bmpr1aa^p.R438H^ | 85,565 | 25 |
| 24 | bmpr1aa^p.R438H^ | 125,047 | 26 |
| 25 | bmpr1aa^p.R438H^ | 94,274 | 26 |

Table S13: Measurements of AV valve area (μm^2^) in adult zebrafish with Tg(fli1a:Gal4FF)^ubs3^; Tg(UAS:bmpr1aa_IRES_EGFP); Tg(7xTCF-Xia.Sia:NLS-mCherry)^ia5^ overexpressing either bmpr1aa^WT^ or bmpr1aa^p.R438H^ within endocardium/endothelium and corresponding fish length.

**REFERENCES**

1. Schunkert, H. *et al.* A Large Pedigree With Valvuloseptal Defects. *Am. J. Cardiol.* **80,** 968–970 (1997).

2. Kong, A. & Cox, N. J. Allele-Sharing Models: LOD Scores and Accurate Linkage Tests. *Am. J. Hum. Genet.* **61,** 1179–1188 (1997).

3. Howe, J. R. *et al.* The prevalence of MADH4 and BMPR1A mutations in juvenile polyposis and absence of BMPR2, BMPR1B, and ACVR1 mutations. *J. Med. Genet.* **41,** 484–491 (2004).

4. Smith, K. A. *et al.* Bmp and Nodal Independently Regulate lefty1 Expression to Maintain Unilateral Nodal Activity during Left-Right Axis Specification in Zebrafish. *PLoS Genet.* **7,** e1002289 (2011).
